# Conference Report: THE AUTOMATIC RADIO FREQUENCY TECHNIQUES GROUP CONFERENCE ON CHARACTERIZATION OF BROADBAND TELECOMMUNICATIONS COMPONENTS AND SYSTEMS Denver, CO June 13, 1997

**DOI:** 10.6028/jres.102.048

**Published:** 1997

**Authors:** Roger B. Marks, Gary D. Alley

**Affiliations:** Electromagnetic Fields Division, Electronics and Engineering Laboratory, National Institute of Standards and Technology, 325 Broadway, Boulder, CO 80303-3328; Lucent Technologies, 1600 Osgood St., N. Andover, MA 01845

## 1. Introduction

While most of the booming consumer and industry demand for data transport is for data *into* the home or office, the “return-path” or “upstream” data requirements are growing rapidly in markets such as telecommuting and video conferences. To satisfy the demand, the major telecommunications players are rushing to establish high bandwidth, two-way digital networks for video, telephony, and Internet to homes and businesses.

One major technology expected to play a key role as a future broadband telecommunications system is two-way digital transmissions over coaxial cable. An advantage of such systems is that much of the infrastructure is currently in place for analog cable television systems. The major upcoming competitors to coaxial systems are based on wireless transmission. What these have in common is radio frequency (RF) technology. Successful deployment relies on RF components and subsystems of exceptionally high performance.

The Automatic Radio Frequency Techniques Group (ARFTG) annually sponsors two conferences on critical topics in RF technology, with a focus on measurement issues. For its 49th Conference, held in Denver, CO on June 13, 1997, ARFTG chose the theme “Characterization of Broadband Telecommunications Components and Systems” to address the critical RF technology issues of broadband communications, needs that have not been directly addressed by other microwave or radio frequency symposia. The conference, which was cosponsored by the Microwave Theory and Techniques (MTT) Society of the Institute of Electrical and Electronics Engineers (IEEE), featured 18 technical talks, 11 poster presentations, and a product exhibition. It proved to be popular, as the registration level of 205 broke the ARFTG record of 168. Attendees came at least 18 nations: Australia, Belgium, China, Finland, France, Germany, Italy, Japan, Korea, the Netherlands, Norway, Canada, Poland, Singapore, Sweden, Taiwan, the United Kingdom, and the United States. Dr. Roger Marks of NIST was the Conference Chair.

## 2. Oral Sessions

The Technical Sessions, under the direction of Conference Technical Program Chair Dr. Gary Alley of Lucent Technologies, included 18 talks and 11 poster presentations. The oral sessions were presented in the traditional ARFTG single-track format. The morning talks focused on coaxial systems and the afternoon on wireless.

### 2.1 Broadband Coaxial Systems

Over the past 7 years, community antenna television (CATV, or cable television) systems have been evolving from one-way transmission of NTSC (U.S. Standard) video signals using long cascades of coaxial amplifiers and directional couplers into bidirectional transmission of NTSC video and digital signals on Hybrid Fiber-Coaxial (HFC) distribution systems. The use of linear lightwave transmitters and receivers has resulted in improved performance for NTSC video transmission but has increased the degradation in digital performance due to laser clipping caused by peaks produced by the broadband multichannel NTSC video waveform. HFC systems being deployed today typically transmit signals from the headend to the home in the 50 MHz to 750 MHz portion of the spectrum while transmitting digital signals from the home to the headend in the 5 MHz to 40 MHz band. The downstream band from 50 MHz to 550 MHz is generally used for up to 83 NTSC video channels while the spectrum from 550 MHz to 750 MHz is reserved for a mixture of digital services including telephony, video telephony, compressed digital video, and Internet access. It has been known for many years that the performance of the upstream portion of these systems, at 5 MHz to 40 MHz, was limited by ingress into the coaxial portion of the system. The sources of this ingress include impulse noise due to lightning and power line currents, intermodulation noise due to the downstream signals in the 50 MHz to 750 MHz band, and coupling of RF broadcast signals into the cable system. The effect of these interfering signals on upstream digital transmission is the subject of current research.

The conference talks on broadband coaxial systems were:

- RF Measurements for Broadband Networks, S. Fluck (Hewlett-Packard Co., Santa Rosa, CA)
  The morning sessions led off with an invited keynote address by Syd Fluck of Hewlett-Packard. Fluck discussed the evolution of CATV systems from the 1960s through the present. The introduction of digital transmission on cable systems resulted in the need to characterize the digital performance of these systems in their commercial environment. Current test sets and methods required to accomplish this task were discussed.
- Proofing and Maintaining Upstream Cable Plant with Digital Signal Analysis Techniques, T. H. Williams (Holtzman Engineering, Longmont, CO)
  Williams discussed the test and maintenance needs of the reverse plant, as well as a set of nontraditional burst-mode tests. These tests used a high speed analog-to-digital converter to capture test and data signals which were then analyzed using a personal computer located at the headend. Examples of data collected and analyzed using these techniques were presented.
- Procedure for Measurement and Characterization of Upstream Channel Noise in CATV Networks, K. Haelvoet, D. De Bal, K. De Kesel, B. Vanlandschoot, and L. Martens (University of Gent, Belgium)
  This paper presented methods for characterizing both ingress and impulse noise in the upstream portion of the system. These methods were independent of the modulation and access techniques used in the systems. Examples were presented using data collected in the field.
- Performance of QAM on a Hybrid Fiber-Coax CATV System, C. Bianchi (Sanders Associates), G. Lentz (Lucent Technologies), and R. Welter (CableVision NY)
  Bianchi discussed the digital system performance of an operational HFC CATV system using measured data from both upstream and downstream 4 and 64 Quadrature Amplitude Modulation (QAM) modems. The resulting performance is found to vary with respect to time, to signal amplitude, and to direction of transmission. In the forward path, the effect of background impairments varies with time and signal power. In the return path, the effect of interferer-like impairments limits the system performance. Statistical bit-error-rate (BER) data were presented along with BER and signal-to-noise (*S*/*N*) data which show the variation in system performance with time.
- Alignment and Maintenance Issues for Upstream Cable Plant, W. Morgan (Hewlett-Packard Co., Santa Rosa, CA)
  Morgan presented a paper which summarized the issues involved with the initial return path alignment and suggestions for the continued maintenance of the return path.
- Characterization of Cable Amplifiers for Broadband Network Applications, J. Steel, A. Parker, and D. Skellern (Macquarie University, Australia)
  Steel presented a paper which discussed the need for characterization of cable network elements for use in digital cable networks. The paper focused on the characterization of nonlinear, frequency-dependent network elements by using a combination of filter blocks and nonlinear blocks. The 2nd and 3rd order distortion performance of CATV amplifiers was presented as an example of the technique.
- Optimal Control of Intermodulation Distortion in Hybrid Fiber Coaxial CATV Systems, G. D. Alley and Y. L. Kuo (Lucent Technologies, N. Andover, MA)
  Alley discussed a technique for minimizing the peak-to-RMS values of the broadband multichannel NTSC video waveform while minimizing both the 2nd and 3rd order distortion products. This was accomplished by optimally controlling the phases of the NTSC video carriers. Both theoretical and experimental results were presented. This paper was selected to receive the conference’s Best Paper Award.
- CATV Tap and Splitter Linearity Improvement for Broadband Information Networks, M. W. Goodwin (Lucent Technologies, N. Andover, MA)
  Goodwin presented an analysis of the source of the intermodulation distortion in HFC CATV systems produced by CATV taps and splitter/combiners, as well as a method for improving the linearity of these devices.

### 2.2 Broadband Wireless Systems

Broadband wireless access is emerging as an alternative method of providing a high capacity digital channel to and from the home. Multichannel Multipoint Distribution Systems (MMDS, or “wireless cable”) have existed for some time, offering up to 33 analog video channels in the 2.150 GHz to 2.682 GHz band. The current direction with MMDS is the introduction of compressed digital video in an effort to increase the capacity of these systems and make them more competitive with cable and satellite video distribution systems. The FCC’s allocation of over 1 GHz of bandwidth between 27.5 GHz and 31.3 GHz has stimulated interest in Local Multipoint Distribution Systems (LMDS). The bandwidth available is expected to provide high capacity two-way wireless services to the home. Wireless systems are physically easier to deploy than wired systems but still present significant challenges to the system provider. Issues such as co-channel adjacent-cell interference and multipath propagation with the resulting finite coherence bandwidth must be addressed.

- LMDS System Design and Performance, W. McKissock and B. Bossard (CellularVision USA, New York, NY)
  The afternoon sessions began with a talk by the originator of LMDS, Bernard Bossard. Bossard discussed the status and design considerations of his company’s LMDS system.
- 28 GHz LMDS System Deployment Considerations, M. S. Shakouri (Hewlett-Packard, Cupertino, CA)
  Shakouri described a system architecture which would provide broadband two-way LMDS service to the home.
- 64 QAM MMDS with Digital Video Compression, M. Frankel (Decathlon Communications, Inc., Englewood, CO)
  Frankel described a proposed implementation of 64 QAM compressed digital video on a 2 GHz MMDS system.
- Conversion Gain Measurement of an LMDS Transmitter, L. R. Hoover and K. W. Burkhart (Texas Instruments, Inc., Dallas, TX)
  Hoover presented a real-time conversion gain measurement technique for an LMDS transmitter utilizing swept scalar analysis and compared the results with a traditional discrete point method.
- Characterization of Miniature Millimeter-Wave Vivaldi Antenna for Local Multipoint Distribution Service, R. N. Simons and R. Q. Lee (NASA Lewis Research Center, Cleveland, OH)
  Simons presented an efficient measurement technique to characterize the input impedance and directional gain of a Vivaldi antenna together with a simple technique to impedance match the antenna to a 50 ohm termination.
- Low Phase Noise Gunn Diode MIC VCOs for Application in Digital Radios, E. M. Godshalk (Redpoint Microwave, Newburg, OR) and V. K. Tripathi (Oregon State University, Corvallis, OR)
  Godshalk presented design information together with measured data for a low phase noise 18 GHz Gunn diode voltage-controlled oscillator.
- Characterizing Components under Large Signal Excitation: Defining Sensible “Large Signal S Parameters”, J. Verspecht, M. Vanden Bossche, and F. Verbeyst (Hewlett-Packard Network Measurement and Description Group, Brussels, Belgium)
  Verspecht presented a black-box measurement technique which characterizes the nonlinear performance of microwave components under periodic large-signal excitation. This technique is based on a vectorial nonlinear-network analyzer, which accurately measures the phase and amplitude of all spectral components of both incident and reflected traveling waves.
- Transition Analyzer-Based Harmonic and Waveform Load Pull Measurements, J. W. Bao, M. Shirokov, C. J. Wei, and J. C. M. Hwang (Lehigh University, Bethlehem, PA)
  Bao presented a new load-pull measurement technique based on a microwave transition analyzer. This technique uses in-situ calibration and large-signal verification to improve measurement accuracy.
- Automated Measurement Procedures of Three-Port and Four-Port Devices on Silicon Wafers, F. Rérat, J.-L. Carbonéro, G. Morin (SGS Thompson Microelectronics, Crolles, France), and B. Cabon (LEMO/ENSERG/INPG, Grenoble, France)
  Rérat described a new method which allows RF measurement and characterization of three and four-port RF integrated circuits on silicon wafers.
- Performance Optimization of Ka-Band MMIC Power Amplifier Using On-Wafer Pulsed Power Test, D. C. Yang, J. M. Yang, P. Nussenbaumer, and M. D. Biedenbender (TRW Inc. RF Product Center, Redondo Beach, CA)
  Yang presented a novel technique which has been developed to optimize power monotithic microwave integrated circuit (MMIC) performance by using on-wafer pulsed power tests. The tests are used to determine the functionality of the device and allows the MMIC chip performance to be optimized through manual bias tuning at the module level.

## 3. Interactive Forum

The Conference included 11 poster papers in an Interactive Forum. While these papers addressed significant problems in ARFTG’s traditional field of microwave measurements, most did not directly address the primary conference theme. These papers were:

- Determination of Bias Dependent Source Resistances in GaAs MESFETs under Cold-FET Conditions, C.-H. Kim, K. S. Yoon, M.-G. Kim, J.-W. Yang, J.-J. Lee, and K.-E. Pyun (Electronics and Telecommunications Research Institute, Taejon, Korea)
- Multiport Network Analyzer Self-Calibration: a New Approach and Some Interesting Results, G. Madonna, A. Ferrero, and U. Pisani (Politecnico di Torino, Italy)
- 60 GHz On-Wafer Noise Parameter Measurements Using Cold-Source Method, M. Lahdes, M. Sipalä, and J. Tuovinen (Millimetre Wave Laboratory of Finland, Espoo, Finland)
- A Programmable Load for Noise Characterization, L. Klapproth, M. Tempel, and G. Boeck (Technical University of Berlin, Germany)
- Improved Measurement Procedure for Extremely Low Noise Figures of FETs in the Frequency Range below 3 GHz, P. Heymann, R. Doerner, and H. Prinzler (Ferdinand-Braun-Institute für Höchstfrequenztechnik, Berlin, Germany)
- The Multi-State Radiometer: A Novel Means for Broadband Noise and Small-Signal Characterization of Microwave Semiconductor Devices, W. Wiatr and M. Schmidt-Szalowski (Warsaw University of Technology, Poland)
- Verification of the Noise Parameter Instrumentation,V. Adamian (ATN Microwave, Inc., N. Billerica, MA)
- Accurate Package Modeling based on S-Parameter Measurements, F. Lin, M. Iyer, H. Ma, K.S. Tan (Institute of Microelectronics, Singapore) and M. Kasashima, J. Shibata, and H. Nakamura (OKI Electric Industry Co, Tokyo, Japan)
- Non-Invasive Dual-Probe Time Domain Measurements of Incident and Reflected Waves on High-Speed Digital Chip Interconnects, A. Barel, Y. Rolain, and A. Cumps (Vrije Universiteit Brussel, Belgium)
- Microwave On-Wafer Measurements with Active Needle Probe Tips, H. Heuermann (Rosenberger Hochfrequenztechnik, Tittmoning, Germany)
- Automatic Testing of a 28 GHz LMDS Down-converter, J. D. King and J. T. McKenna (M/A-COM Inc., Lowell, MA)

## 4. Joint Session on Crosstalk

On Thursday, June 12, ARFTG cosponsored a session of the 1997 IEEE MTT-S International Microwave Symposium. The session, entitled “Crosstalk, Coupling, and Multiconductor Transmission Line Characterization,” was organized and chaired by Dr. Dylan F. Williams of NIST and co-chaired by Dr. Joy Laskar of the Georgia Institute of Technology. This session provided a forum for the latest developments in the theory and measurement of crosstalk and coupling. The session began by focusing on measurements for the characterization of lossy coupled multiconductor transmission lines. It also featured theoretical treatments of multiconductor lines, coupling between devices, and stimulation of multi-mode systems. The agenda was:

- An Accurate Determination of the Characteristic Impedance Matrix of Symmetrical Coupled Lines on Chips Based on High Frequency S-Parameter Measurements, T. M. Winkel (IBM Deutschland Ent. GmbH, Boeblingen, Germany), L. Dutta (Siemens, Munich, Germany), and H. Grabinski (LFI, University of Hannover, Germany)
- Embedded Multiconductor Transmission Line Characterization, D. F. Williams (NIST, Boulder, CO)
- Characterization of Multiconductor Coupled Lines from Multiport TDR Measurements, A. Tripathi and V. K. Tripathi (Oregon State University, Corvallis, OR)
- Experimental Circuit Model Generation of Non-Uniform Coupled Multi-Conductor Structures, S. Sercu and L. Martens (University of Gent, Belgium)
- Waveform Relaxation Synthesis of Time-Domain Characteristic Model of Coupled Transmission Lines from FDTD Simulation, Q.-X. Chu, F.-Y. Chang (Chinese Univ. of Hong Kong)
- Excitation of the Parasitic Parallel-Plate Line Mode at Coplanar Discontinuities, K. Beilenhoff (TH Darmstadt, Germany) and W. Heinrich (Ferdinand-Braun-Institut, Berlin, Germany)
- Investigation of MMIC Inductor Coupling Effects, M. Werthen, I. Wolff (IMST, Kamp Lintfort, Germany), R. Keller, and W. Bischof (Bosch Telekom GmbH, Backnang, Germany).

## 5. Summary

Broadband telecommunications systems are undergoing rapid development and will soon begin to make a significant thrust into consumer and industry markets. Advances in radio frequency technology are critical to the cost and timing of this potential economic and sociological revolution.

## 6. Proceedings

The 49th ARFTG Conference Digest, which includes 36 papers in 251 pages, was distributed at the conference. Ordering information is available on the ARFTG web site or from the ARFTG Executive Secretary (+1–602–839–6933).

## 7. Future Conferences

The 51st ARFTG Conference will be held in Baltimore on Friday, June 12, 1998 in conjunction with the 1998 IEEE MTT-S International Microwave Symposium. The meeting topic is “Characterization of Spread Spectrum Components and Systems.” In order to continue its focus on broadband telecommunications, ARFTG will cosponsor a Joint Session with the 1998 IEEE MTT-S International Microwave Symposium on the topic “Broadband Telecommunications Systems” during the week of June 8 in Baltimore. A second Joint Session will cover “Digital Interconnection Techniques and Characterization at GHz Frequencies.”

In addition to its odd-numbered conferences in the spring, ARFTG presents an even-numbered conference each fall during the week after Thanksgiving. The 50th ARFTG Conference, on “Measurement Techniques for Digital Wireless Applications,” will take place on December 4–5, 1997 at the Benson Hotel in Portland, OR. ARFTG and NIST will also present their fourth annual Microwave Measurements Short Course on December 2–3.

## 8. More Information

More information on ARFTG and its conferences is available on the ARFTG Web Site at *http://www.arftg.org.*

